# Behavioral, neurotransmitter and transcriptomic analyses in male and female *Fmr1* KO mice

**DOI:** 10.3389/fnbeh.2024.1458502

**Published:** 2024-09-06

**Authors:** Deirdre M. McCarthy, Cynthia Vied, Mia X. Trupiano, Angeli J. Canekeratne, Yuan Wang, Christopher Schatschneider, Pradeep G. Bhide

**Affiliations:** ^1^Department of Biomedical Sciences, Florida State University College of Medicine, Tallahassee, FL, United States; ^2^Center for Brain Repair, Florida State University College of Medicine, Tallahassee, FL, United States; ^3^FSU Institute for Pediatric Rare Diseases, Florida State University College of Medicine, Tallahassee, FL, United States; ^4^Translational Science Laboratory, Florida State University College of Medicine Tallahassee, FL, United States; ^5^Program in Neuroscience, Florida State University, Tallahassee, FL, United States; ^6^Department of Psychology, College of Arts and Sciences, Florida State University, Tallahassee, FL, United States

**Keywords:** hyperactivity, nest building, impulsivity, GABA, glutamate, substance dependence

## Abstract

**Introduction:**

Fragile X syndrome is an inherited X-linked disorder associated with intellectual disabilities that begin in childhood and last a lifetime. The symptoms overlap with autism spectrum disorder, and the syndrome predominantly affects males. Consequently, FXS research tends to favor analysis of social behaviors in males, leaving a gap in our understanding of other behavioral traits, especially in females.

**Methods:**

We used a mouse model of FXS to analyze developmental, behavioral, neurochemical, and transcriptomic profiles in males and females.

**Results:**

Our behavioral assays demonstrated locomotor hyperactivity, motor impulsivity, increased “approach” behavior in an approach-avoidance assay, and deficits in nest building behavior. Analysis of brain neurotransmitter content revealed deficits in striatal GABA, glutamate, and serotonin content. RNA sequencing of the ventral striatum unveiled expression changes associated with neurotransmission as well as motivation and substance use pathways. Sex differences were identified in nest building behavior, striatal neurotransmitter content, and ventral striatal gene expression.

**Discussion:**

In summary, our study identified sex differences in specific behavioral, neurotransmitter, and gene expression phenotypes and gene set enrichment analysis identified significant enrichment of pathways associated with motivation and drug reward.

## Introduction

Fragile X syndrome (FXS) is an inherited pediatric rare disorder that causes a range of developmental disabilities, including delayed speech and language development, intellectual disability, anxiety, attention deficit, hyperactivity, as well as communication and social deficits (Hagerman et al., 2017). Physical features associated with FXS include hypotonia, flat feet, hyperextensible joints, and macroorchidism. The prevalence of FXS is higher in males than in females. It affects approximately 1:4,000 males and 1:8,000 females in the general population (Hunter et al., 2014).

FXS is the result of a mutation in the fragile x messenger ribonucleoprotein 1 (*FMR1*) gene. The mutation expands a DNA segment, known as the CGG triplet repeat in the 5′ untranslated region of *FMR1* beyond its normal range of 5 to 40 repeats. The full mutation (>200 repeats) results in hypermethylation and silencing of the *FMR1* gene and a complete loss or a significant downregulation of the fragile x messenger ribonucleoprotein (FMRP). FMRP is an RNA-binding protein that regulates translation of messenger RNA and serves critical functions in the developing and mature nervous system (Hagerman et al., 2017).

The *Fmr1* knockout (KO) mouse was developed and characterized by Bakker et al. (1994). It shows changes in biochemical (Dahlhaus and El-Husseini, 2010), electrophysiological (Rais et al., 2018; Lovelace et al., 2018), neuropathological (Greco et al., 2011), and dendritic spine morphology (Irwin et al., 2000) features, many of which are consistent with FXS. Despite considerable variability in phenotypes (Paradee et al., 1999; Spencer et al., 2011) the *Fmr1* KO mouse has contributed to major advances in our understanding of the behavioral, molecular, and synaptic changes associated with the loss of *Fmr1* (Kazdoba et al., 2014; Yan et al., 2005; Hebert et al., 2014; Spencer et al., 2005; Yuskaitis et al., 2010; Rotschafer et al., 2012; Schaefer et al., 2017; Gurney et al., 2017; Gantois et al., 2017; Gomis-Gonzalez et al., 2016; Dolan et al., 2013; Jiang et al., 2022).

Traditionally FXS research has focused on men and boys because of the higher rates of prevalence and greater severity of symptoms in males (Hartley et al., 2011). However, FXS in females presents with a variety of challenges including a high frequency of avoidant behavior, shyness, mood disorders, and learning disabilities (Hartley et al., 2011; Murphy and Abbeduto, 2007). Moreover, FXS research tends to focus on cognitive and social behaviors because of symptom overlap between FXS and autism spectrum disorder (Smith et al., 2012; Kaufmann et al., 2004; Roberts et al., 2009; McDuffie et al., 2012; Niu et al., 2017). However, FXS is associated with changes in motivated behaviors including drug reward (Fish et al., 2013; Smith et al., 2014), although these behaviors traditionally have not been the focus of FXS research.

Since a side-by-side analysis of male and female *Fmr1* KO mice could offer further insights into sex differences in the neurobiology of FXS, we examined behavioral, neurotransmitter, and changes in gene expression in both sexes of *Fmr1* KO mice. For the gene expression analysis, we focused on the ventral striatum because it is at the crossroads of multiple behaviors associated with the limbic system, and ventral striatal transcriptome had not been analyzed previously in the *Fmr1* KO mouse. Our data demonstrate significant sex-specific changes in behavioral and neurotransmitter phenotypes as well as gene expression in the ventral striatum.

## Methods

### Animals

Eight-week-old male and female C57BL/6J^1^ and *Fmr1* KO^2^ mice were purchased from Jackson Laboratories (Bar Harbor, ME). The line was maintained by breeding hemizygous male *Fmr1* mice with homozygous female *Fmr1* mice. Wild-type (WT) mice of the same strain were used as controls, consistent with the design of experiments using this mouse model published previously (Kat et al., 2022). Five cohorts of mice were generated to provide sufficient numbers for all the analyses. Mice were maintained in a temperature-controlled environment with a 12 h/12 h light/dark cycle with food and water available *ad libitum*. The day of birth was designated P0. Litter size was recorded on P0, and entire litters were weighed on P0, P7, P14, and P21 for offspring body weight measurements. Mice were weaned on P21 per standard husbandry procedures. All the experimental procedures were approved by the Animal Care and Use Committee at Florida State University and were in full compliance with the NIH Guide for the Care and Use of Laboratory Animals.

### Developmental milestones

Mice were monitored throughout the pre-weaning period for body weight, bilateral external ear detachment, body fur appearance, and eye-opening and tested for neuromotor skills such as righting reflex, cliff avoidance, and negative geotaxis. Analysis of body weight and physical features did not discriminate between males and females. For the assessment of neuromotor skills, male and female mice were analyzed separately, and sex was an independent variable. In all cases, 4–5 litters were used, and litter was used as “*n*” for statistical analyses.

Offspring were tested for righting reflex beginning at P2 by placing them in a supine position on a flat surface and measuring the time taken to “right” onto all four paws. This test was repeated daily, with one trial per day, until the mice met the criterion of righting within 3 s on two consecutive days.

Cliff avoidance reflex was tested beginning on P6 by placing the mouse on a flat Plexiglass surface raised to a height of 18 cm above the workbench. The pup was placed with its front paws and snout over the edge of the Plexiglass, and the time taken to turn 180° away from the cliff (edge) was recorded. The test was repeated daily until a latency of 6 s or less (criterion) was met on two consecutive days.

Negative geotaxis was assessed beginning on P6 by placing the pups head-downward on a 30° mesh incline and the time to turn 180° and reverse orientation to face upward was measured. Mice were tested once daily until a latency of 6 s or less (criterion) was reached for two consecutive days.

If the mice did not complete any of the neuromotor skill assessments within 30 s, or if they fell off the test apparatus at any time the tests were terminated, and the mice were assigned the full score of 30 s and returned to their home cages. The order of testing on each day was: Cliff avoidance, negative geotaxis, and righting reflex. A rest period of at least 15 min was used between tests (Cole et al., 2012).

### Behavioral analyses in adult mice

Behavioral analyses were performed in adult (2–3-month-old) male and female WT and *Fmr1* KO mice during the lights-off period when mice are naturally more active. A dim red light was used for ambient illumination. Mice from each genotype and sex were tested on each test day in parallel sessions. The experimenter was blinded to the identity of the mice. Each maze or test apparatus was cleaned with 1.6% Quatracide before testing and between trials to eliminate odor cues.

Mice were handled by the experimenter for at least 2 days, 2 min per day, for 1 week before the beginning of behavioral analyses. Prior to the commencement of each behavioral test, 30 min of habituation was used to permit acclimation to the testing room environment. The *n* used was 6–9 per genotype per sex for all behaviors except for nest building where the *n* was 7–15. When a mouse completed more than one behavioral test, we waited 1 week before administering the next behavioral test. Given the extensive battery of behavioral analyses performed, 3 cohorts of mice were used in total.

### Spontaneous locomotor activity

Locomotor activity was measured in individual testing chambers equipped with photobeam motion sensors (Photobeam Activity System; San Diego Instruments), which create a 3-dimensional grid (5.4 cm spacing) of infrared beams enveloping the entire cage (Zhu et al., 2017; McCarthy et al., 2018; McCarthy et al., 2020). As the mouse moved along the x- and y-axes, the number of breaks in the infrared beams was recorded. Each consecutive beam break was scored as an ambulatory event. The total ambulatory activity was analyzed over a period of 14 h which included an initial 2 h “lights-on” period (exploratory activity) and a subsequent 12 h “lights-off” period (spontaneous locomotor activity).

### Approach-avoidance behavior

An elevated plus maze (EPM) with two open and two closed arms (50 cm × 10 cm each) with 40 cm high walls was used (Med Associates, Inc., St. Albans, VT). The time spent in the open and closed arms as well as the number of entries into the open and closed arms were recorded over a 5 min test period (Martin et al., 2020).

### Object-based attention

The test apparatus consisted of an exploration chamber and a test chamber separated by a sliding door. The test consisted of 3 sessions: habituation (day 1), training (day 2) and test (day 3). During the habituation session, each mouse was placed in the empty exploration and test chambers for 5 min each. During the training session, each mouse explored 5 uniquely shaped objects for 5 min in the exploration chamber. During the test session, each mouse interacted with one previously explored familiar object and one previously unexplored novel object in the test chamber for 3 min. A recognition index for the test session was calculated using the formula: TN ÷ (TF + TN) × 100, where TF = Time spent exploring the Familiar objects, and TN = Time spent exploring the Novel objects (McCarthy et al., 2018; McCarthy et al., 2020). We included in the analysis only those mice that spent at least 20 s with both objects during the test session.

### Spatial working memory

A custom-built clear Plexiglass maze with 3 equal-sized arms arranged in the shape of the letter Y (Y-maze; each arm = 35 cm long × 6 cm wide × 10 cm high; McCarthy et al., 2018; McCarthy et al., 2020) was used. Unique visual cues were placed on the outer walls of each arm. A letter code (A, B, or C) was assigned to each arm. The mouse was placed at the end of a randomly chosen arm. During a 6 min test period, the sequence of arm entries, and the total number of arm entries were recorded. A “spontaneous alternation” is a set of 3 nonrepeating consecutive arm choices (e.g., ABC, BCA, CBA but not ABB, CCB, BAA, etc.). An alternation index (a measure of spatial working memory) was calculated as follows: number of alternations ÷ (number of entries − 2) × 100.

### Motor-impulsivity

A cliff avoidance reflex (CAR) test was used (McCarthy et al., 2020; Zhang et al., 2018). Mice were placed at the center of a raised platform (20 cm in diameter) supported on a plastic rod (50 cm in height) resembling a barstool. The number of falls/jumps from the platform over 30 min was recorded for each mouse. If a mouse fell/jumped off the platform, it was gently picked up and returned to the center of the platform. A mouse that remained on the platform received a CAR score of 1 whereas a mouse that fell from the platform (regardless of the number of falls) received a score of 0.

### Social dominance

The apparatus consisted of a Plexiglass tube approximately 17 inches long and 1 inch internal diameter (Maze Engineers, Skokie, IL; Spencer et al., 2005). The test included a 3-day training period during which the mice were habituated daily to the tube for 2 min. On test day one WT mouse and one *Fmr1* KO mouse (age- and sex-matched) were placed headfirst at opposite ends of the tube and simultaneously released into the tube. Once both mice arrived at the center of the tube, the divider separating the mice was raised. The “match” lasted 2 min and ended when one mouse completely retreated from the tube. The mouse that remained in the tube is designated the winner (score = 1), and the mouse that retreated is the loser (score = 0). A match that lasted greater than 2 min was not scored, as 2 min was the cut-off time for this test. Each mouse competed in three matches in total.

### Nest building

Group-housed mice were removed from their cages and housed individually in new cages (home cage) for a week and then nest building was examined in the home cage (7.25″ W × 11.5″ D × 5″ H). Four days later, the test was repeated in a novel environment represented by a new, larger mouse breeder cage (10.5″ W × 19″ D × 6.25″ H) placed in a new room (Carreno-Munoz et al., 2018). During the tests, mice were provided with a single 5 cm^2^ condensed cotton nestlet, which the mice tore up to build nests. The quality and condition of the nests were scored on a 5-point scale at 30 min intervals for 5 h, as follows. Score 1: The nestlet material was essentially untouched (>95% intact) and no nest was built. Score 2: The nestlet was partially torn up (50–95% remaining intact) and no nest was built. Score 3: The majority of the nestlet (but <90%) was shredded and evidence of attempts at nest building could be seen. Score 4: >90% of the nestlet was torn up and the material was used to construct a nest, which was flat and not dome-shaped. Score 5: Most of the nestlet (>90%) was torn up and a perfect dome-shaped nest with a crater and a wall could be seen. The nest wall was taller than the mouse along >50% of the nest circumference. The test period of 5 h was chosen for home cage nesting because WT mice achieved the maximum nest score by 5 h. In the novel environment, WT mice required more time to achieve the maximum nest score, therefore, we monitored the mice for an additional 3 h (a total of 8 h).

### Tissue collection

Following the behavioral analyses, the mice were weighed, euthanized with isoflurane anesthetic overdose, decapitated, and the brains dissected from the skull. The dorsal and ventral striatum, prefrontal cortex, medial prefrontal cortex, and hippocampus were micro-dissected based on anatomical landmarks from both hemispheres (McCarthy et al., 2018). Samples from the 2 hemispheres were pooled, weighed, and immediately frozen with liquid nitrogen. Testes and adrenal glands were removed and weighed, and the weights were converted to percentages of body weight for each mouse. The *n* used was 4–7 per genotype per sex.

### High-performance liquid chromatography

Tissue samples were shipped to the Neurochemistry Core at Vanderbilt University (Nashville, TN), where they were homogenized, and the protein concentration was estimated in each sample as previously described (McCarthy et al., 2018). Tissue concentrations (ng/mg protein) of dopamine, norepinephrine (NE), gamma-aminobutyric acid (GABA), glutamate, and serotonin were analyzed.

### RNA sequencing

Samples of the ventral striatum were processed for RNA extraction as previously described (Qiagen RNeasy mini kit McCarthy et al., 2018). A next-generation sequencing library was prepared for each sample using the Illumina TruSeq stranded mRNA library kit. Libraries were barcoded for multiplexing with IDT for Illumina unique dual indexes. Each multiplexed library was sequenced on an Illumina NovaSeq 6000 as a 150 base pair paired-end sequencing run. Adapter trimming was performed as part of individual library demultiplexing. Quality Control analysis of each library was performed using fastQC.^3^ Illumina RNA-Seq Alignment (Version 2.0.2) was used, including STAR Aligner (Dobin et al., 2013) to align and map sequencing reads to the mouse genome (genome release UCSC mm10) and to generate read counts, which were used as a measure of abundance for differential gene expression analysis. DESeq2 (Love et al., 2014) was used to determine statistically significant differentially expressed genes (a false discovery rate, FDR, of <0.05 was used) and to generate a principal component analysis (PCA) plot. The analysis yielded a list of mRNAs present at significantly different levels between the WT and *Fmr1* KO mice and provided a measure of confidence in each difference. Genes with a statistically significant differential expression were further analyzed by Kyoto Encyclopedia of Genes and Genomes (KEGG) pathway analysis and phenotype set enrichment analyses using Webgestalt (Zhang et al., 2005; Wang et al., 2013) to establish possible functional roles (Brown et al., 2015; Darkazalli et al., 2017; Jones et al., 2022; Vied et al., 2016; Vied et al., 2014). Benjamini–Hochberg procedure was used as the multiple test adjustment parameter against the mouse genome as a reference for enrichment and a *p*-value of <0.05 was considered significant. The heat map was generated using Morpheus (Broad Institute; https://software.broadinstitute.org/morpheus). All data are available in the NCBI Gene Expression Omnibus (Accession number GSE275170).^4^ Data was collected from four male and female WT and *Fmr1* KO mice per genotype per sex. One outlier (*Fmr1* KO Male 2) was excluded from the analysis based on the PCA generated during the DESeq2 analysis.

### Quantitative PCR

Tissue samples were processed for RNA extraction as previously described (Qiagen; RNeasy mini kit McCarthy et al., 2018). Reverse transcription reactions were performed using the SuperScript III cDNA synthesis kit (Thermo Fisher Scientific; 18080-044). Primer sequences for 18 s (Thermo Fischer Scientific, Hs Hs99999901_s1) and *Fmr1* (Thermo Fischer Scientific, Mm01339582_m1) were chosen based on previously published data (McCarthy et al., 2018; Zhao and Usdin, 2014). Quantitative real-time PCR was performed in a StepOne Plus Thermocycler (Thermo Fisher Scientific) using Taqman PCR Master Mix (Thermo Fisher Scientific; 4369016) through 50 PCR cycles (95°C for 30 s, 57°C for 60 s, 72°C for 90 s). *Fmr1* mRNA expression was normalized to 18 s using the Delta- Delta Ct method. Fold change was calculated for each sex separately using the WT values. The *n* used was 3 mice per genotype per sex.

### Western blot

From each homogenized striatal tissue sample, 30 μg protein was loaded on a 10% SDS-PAGE gel and the separated proteins were transferred to the PVDF membrane. The membranes were incubated with rabbit anti-FMRP monoclonal antibody (Cell Signaling Technology, #7104; 1:1,000; 80 kDa), and mouse β-tubulin antibody (loading control; Sigma Aldrich, #T8328; 1:5,000; 50 kDa). Immunoreactive bands were detected using goat anti-mouse IRDye 800 (Li-Cor, #926-32210; 1:20,000) and anti-rabbit IRDye 680 (Li-Cor, #926-68071; 1:20,000). The signals were captured using Odyssey CLx Imaging System (Li-Cor). The n used was 2 mice per genotype per sex.

### Statistical analysis of behavioral and neurotransmitter data

A series of mixed model ANOVA followed by *post hoc* contrasts, where appropriate, using the Benjamini–Hochberg linear step up procedure to control for type I error were used to analyze developmental milestones, exploratory behavior (EPM and home cage activity) locomotor activity, nest building behavior, spatial working memory, and object-based attention (OBA). A binary logistic regression model was used to analyze the cliff avoidance reflex score. A negative binomial distribution was used to analyze the number of falls from the platform in the cliff avoidance reflex assay. Binomial logistic regression was used to analyze the tube test scores to determine whether scores were significantly different from an outcome expected by chance (50:50 win-lose outcome). A two-tailed student’s *t*-test was used to analyze testes weight where differences between only two groups were analyzed. Statistical analyses were performed using SAS/STAT 9.4 (SAS, Cary, NC). When a significant effect of genotype was observed without a significant effect of sex, data combined from male and female mice for each genotype (WT and KO) are shown in the Figures. Post-contrast analyses were performed only when a statistically significant interaction was found. Graphs were prepared using Prism 10.1.2 (GraphPad Prism, San Diego, CA).

## Results

### Developmental milestones in preweaning mice

Bodyweights at P0, P7, P14, and P21 were recorded and analyzed for entire litters without separating male and female offspring. The data did not reveal significant effects of genotype (*F*_(1,27)_ = 0.64; *p* > 0.05), or genotype x age interaction (*F*_(3,27)_ = 1.29; *p* > 0.05). However, there was a significant effect of age (*F*_(4,27)_ = 159.62; *p* < 0.001) reflecting bodyweight gain during development in both *Fmr1* KO and WT mice. The analyses of postnatal days on which the external ears detached, body fur appeared, or eyes opened did not show significant effects of genotype (student’s *t*-test, *p* > 0.05; Table 1).

**Table 1:** Litter metrics and developmental milestones (mean ± SE).

	WT	*Fmr1* KO
Litter size at birth	6.0 ± 0.8	5.7 ± 0.7
Postnatal weight (g) on:		
Postnatal day 0 (day of birth)	1.1 ± 0.2	1.2 ± 0.1
Postnatal day 7	4.3 ± 0.3	3.9 ± 0.1
Postnatal day 14	7.1 ± 0.4	7.0 ± 0.5
Postnatal day 21	9.2 ± 0.4	10.1 ± 0.5
Ear detachment (postnatal day)	4.0 ± 0.0	4.4 ± 0.2
Fur appearance (postnatal day)	7.0 ± 0.0	7.0 ± 0.0
Eye opening (postnatal day)	14.0 ± 0.0	14.3 ± 0.2
Acquisition of:		
Righting reflex (postnatal day)	8.8 ± 0.57	9.4 ± 0.55
Negative geotaxis (postnatal day)	12.0 ± 0.8	9.8 ± 0.8
Cliff avoidance (postnatal day)	13.4 ± 1.1	12.0 ± 1.0

Litter size on the day of birth and developmental milestones during the pre-weaning period were analyzed in *Fmr1* KO and WT mice. A mixed model ANOVA was used to test the statistical significance of the effects of genotype, age, sex, and interactions. A two-tailed students’ *t*-test was used for the analysis of statistical significance in the case of physical features. There were no differences between the WT and *Fmr1* KO mice in any of the measurements.

There was no significant effect of genotype on righting reflex (*F*_(1,12)_ = 0.53; *p* > 0.05), cliff avoidance (*F*_(1,12)_ = 0.87, *p* > 0.05), or negative geotaxis (*F*_(1,12)_ = 3.98, *p* > 0.05; Table 1 and Supplementary Figure S1). There was no significant effect of sex or sex x genotype interaction on any of the neuromotor skills (*p* > 0.05).

### Spontaneous locomotor activity

**Figure 1:**
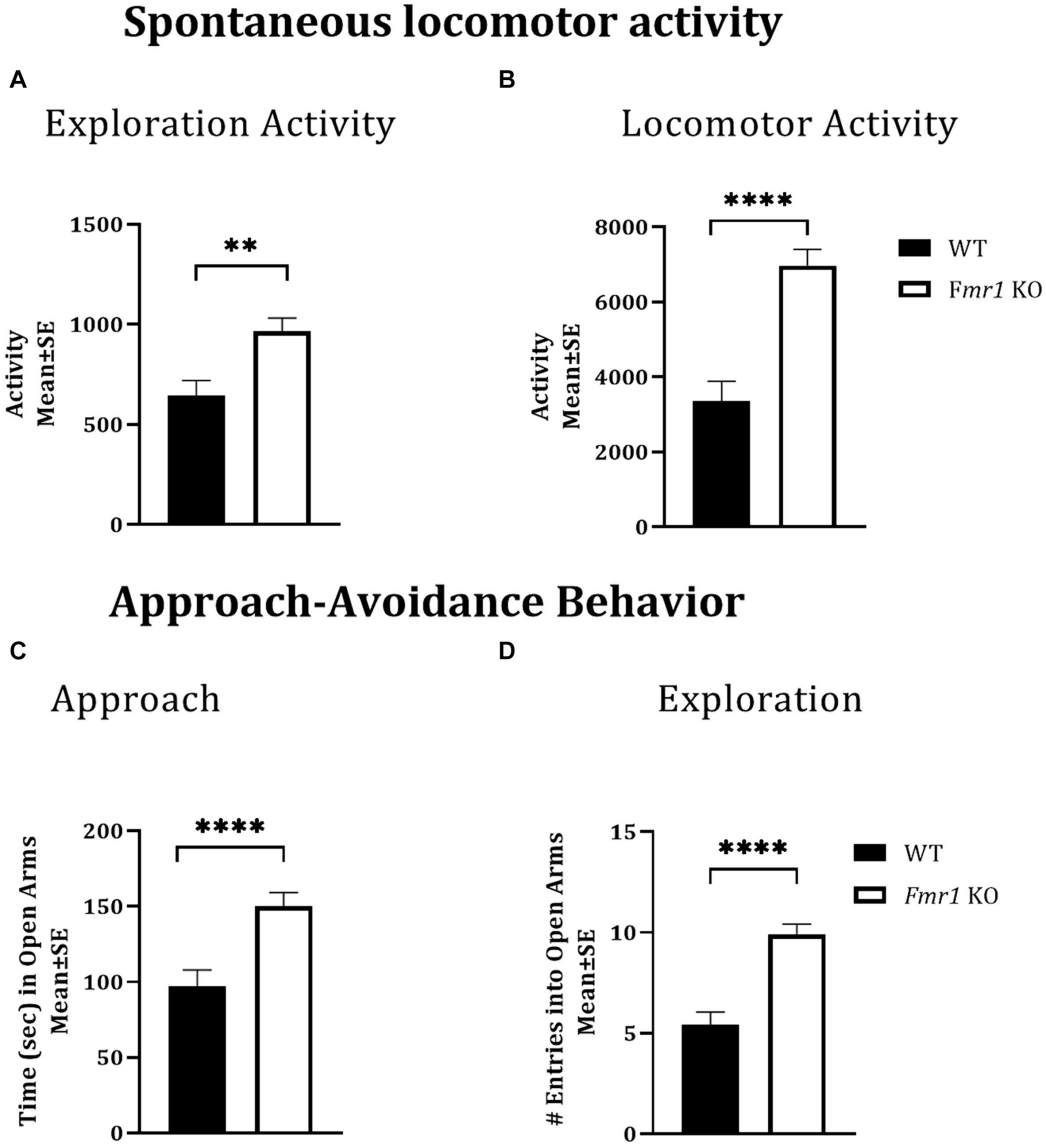
Exploratory activity, spontaneous locomotor activity, and approach behavior in WT and *Fmr1* KO mice. *Fmr1* KO mice showed significant increases in locomotor activity during the lights-on period (**A**; exploratory activity in a novel environment) and the lights-off period (**B**; spontaneous locomotor activity) compared to WT mice. *Fmr1* KO mice spent significantly more time in the open arms of the elevated plus maze (**C**; approach behavior) and made significantly greater number of entries into the open arms of the maze (**D**; exploration) compared to WT mice. A mixed model ANOVA showed a significant effect of genotype (asterisks) and no significant effect of sex or genotype x sex interaction Therefore, data from male and female mice from each genotype were combined to illustrate the differences between genotypes. ^**^p < 0.01 and ^****^p < 0.0001, *n* = 6–9 per genotype per sex.

Locomotor activity was analyzed over a period of 14 h which included an initial 2 h “lights-on” period (exploratory activity) and a subsequent 12 h “lights-off” period (McCarthy et al., 2018; McCarthy et al., 2020). There was a significant effect of genotype during the initial 2 h lights-on period (i.e., exploratory activity; Figure 1A: asterisks; *Fmr1* > WT; *F*_(1,25)_ = 10.26; *p* < 0.01) and during the 12 h lights off period (spontaneous locomotor activity; Figure 1B; asterisks; *Fmr1* > WT; *F*_(1,25)_ = 27.56; *p* < 0.001). There was no significant effect of sex (exploratory activity: *F*_(1,25)_ = 3.87; *p* > 0.05; spontaneous locomotor activity: *F*_(1,25)_ = 1.24; *p* > 0.05) or sex x genotype interaction (exploratory activity: *F*_(1,25)_ = 0.01; *p* > 0.05; spontaneous locomotor activity: *F*_(1,25)_ = 0.27; *p* > 0.05). Thus, both male and female *Fmr1* KO mice were more exploratory upon placement in the testing environment and were hyperactive in the familiar environment (Figures 1A,B).

### Approach-avoidance behavior

The elevated plus maze was used to test approach-avoidance behavior (Martin et al., 2020). Time spent in the open arms of the maze was taken to represent approach whereas time spent in the closed arms to represent avoidance. There was a significant effect of genotype on the time spent in the open arms of the maze (Figure 1C: asterisks; *Fmr1* > WT; *F*_(1,25)_ = 14.45; *p* < 0.001) and on the number of open arm entries (Figure 1D: asterisks; *Fmr1* > WT; *F*_(1,25)_ = 30.45; *p* < 0.001). There was no significant effect of sex (Time in open: *F*_(1,25)_ = 0.08; *p* > 0.05; entries: *F*_(1,25)_ = 2.14; *p* > 0.05) or genotype x sex interaction (Time in open: *F*_(1,25)_ = 1.66; *p* > 0.05; entries: *F*_(1,25)_ = 0.19; *p* > 0.05), suggesting impaired approach-avoidance balance in favor of approach and novelty seeking in both male and female *Fmr1* KO mice.

### Motor impulsivity

**Figure 2:**
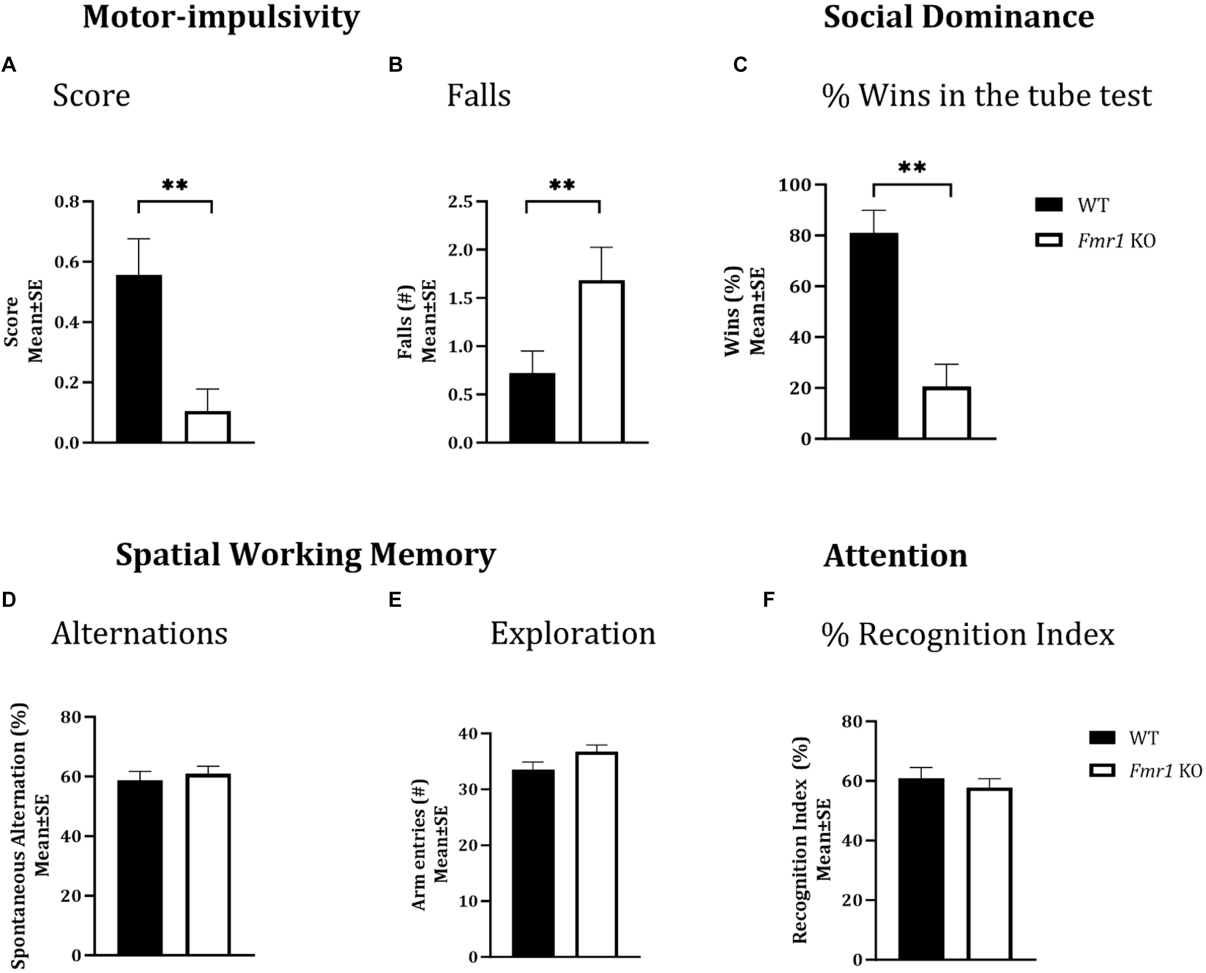
Motor-impulsivity, social dominance, spatial working memory and object based attention in WT and *Fmr1* KO mice. *Fmr1* KO mice had a significantly lower cliff avoidance reflex score (**A**; binary logistic regression) and a significantly greater number of falls from the platform (**B**; negative binomial distribution) compared to WT mice. *Fmr1* KO mice had significantly fewer wins compared to WT mice in the tube test of social dominance (**C**; binomial logistic regression). Significant effects of genotype (asterisks) were found for all three measures **(A–C)**, but the effect of sex was not significant. Spontaneous alternation index, which is a measure of spatial working memory **(D)**; the number of arm entries in the Y-maze (measure of maze exploration; **E**), or recognition index (unit of measure for attention in the object-based attention task; **F**) did not show significant effects of genotype, sex of genotype x sex interaction. Since genotype produced significant effects on some behaviors and sex did not produce significant effects on any measures, data from male and female mice from each genotype were combined to illustrate the differences between genotypes. ^**^p < 0.01; *n* = 6–9 per genotype per sex.

A cliff avoidance reflex test was used to test motor impulsivity. There was a significant effect of genotype on cliff avoidance reflex score (Figure 2A; *Fmr1* > WT; *χ*^2^_(1,37)_ = 7.03; *p* < 0.01). The *Fmr1* KO mice were 10.7 times more likely to fall off the platform than WT mice. There was also a significant effect of genotype on the number of falls from the platform (Figure 2B; *Fmr1* > WT; *χ*^2^_(1,37)_ = 7.41; *p* < 0.01). The effect of sex was not significant for either measure (score: *χ*^2^_(1,37)_ = 0.002; *p* > 0.05; falls: *χ*^2^_(1,37)_ = 0.05; *p* > 0.05) suggesting that motor impulsivity was observed in both male and female *Fmr1* K0 mice.

### Social dominance

A tube test was used to examine social dominance. *Fmr1* KO mice won significantly fewer matches against sex and age-matched WT mice (Figure 2C; *Fmr1* < WT; *χ*^2^_(1,78)_ = 3.02; *p* < 0.01). The *Fmr1* KO mice were 14.3 times more likely to lose a match than WT mice. The effect of sex was not significant (*χ*^2^_(1,78)_ = 0.80; *p* > 0.05) indicating that *Fmr1* KO mice, regardless of sex had a deficit in social dominance behavior.

### Nest building

**Figure 3:**
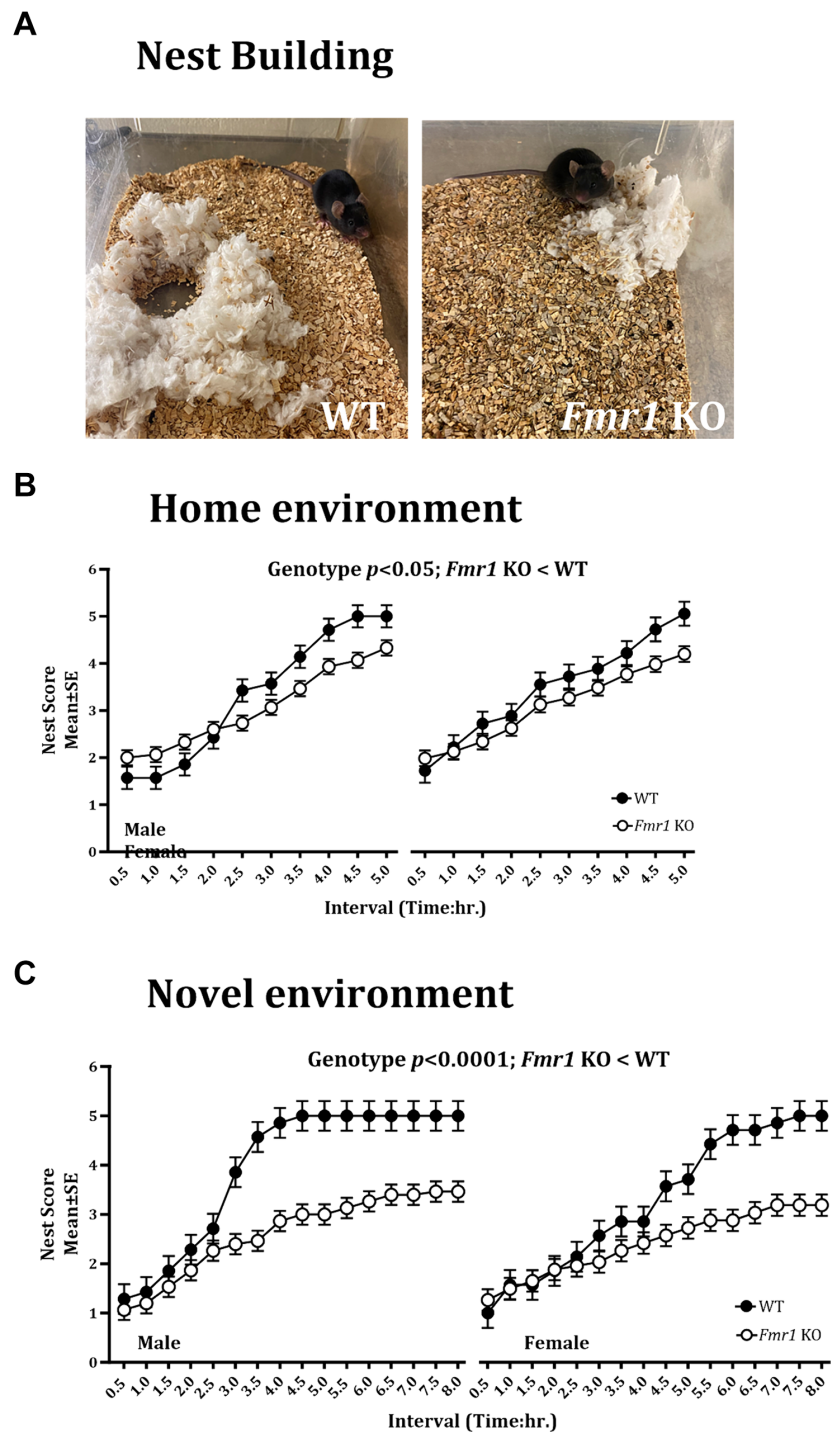
Nest building deficit in male and female *Fmr1* KO mice in the home and novel environments. Representative images of nests built by WT and *Fmr1* KO mice (**A**; novel environment). The *Fmr1* KO mice received significantly lower nest scores compared to the WT mice in the home **(B)** and the novel environment **(C)**. A mixed model ANOVA showed a significant effect of genotype under both conditions. Sex differences were present only in the novel environment. Since genotype, sex, and sex x genotype showed significant effects, data from male and female mice are shown separately for each genotype. *n* = 7–15 per genotype per sex.

Nest building was examined in the home environment and 4 days later, the test was repeated in a novel environment. We found a significant effect of genotype on the nest building score in the home cage (Figure 3B; *Fmr1* KO < WT: *F*_(1,20)_ = 7.54; *p* < 0.05). There was no significant effect of sex (*F*_(1,18)_ = 1.93; *p* > 0.05) or sex x genotype interaction (*F*_(1,18)_ = 0.77; *p* > 0.05) in the home environment. In the novel environment, there was a significant effect of genotype (Figure 3C; *Fmr1* KO < WT: *F*_(1,20)_ = 25.07; *p* < 0.0001), sex (female < male: *F*_(1,18)_ = 61.65; *p* < 0.0001) and genotype x sex interaction (*F*_(1,18)_ = 17.73; *p* < 0.001) on nest building score.

Thus, there were significant effects of genotype on the nest building score in the home and the novel environments (*Fmr1* KO < WT). Sex differences (female < male) and sex x genotype interaction were present only in the novel environment.

An example of a fully formed nest, which was observed typically in WT mice in the novel environment at the end of the 5 h. observation period is shown alongside a flat, poorly formed nest observed typically in *Fmr1* KO mice housed in the novel environment at the end of the 5 h. period (Figure 3A).

### Spatial working memory

A Y-maze was used to test spatial working memory, and the spontaneous alternation index was the test parameter (McCarthy et al., 2018; McCarthy et al., 2020). There was no significant effect of genotype (*F*_(1,25)_ = 0.31; *p* > 0.05), sex (*F*_(1,25)_ = 3.48; *p* > 0.05), or genotype x sex interaction (*F*_(1,25)_ = 0.02; *p* > 0.05) on spontaneous alternations, suggesting that neither male nor female mice showed significant deficits in spatial working memory (Figure 2D). Similarly, the total number of arm entries did not show significant effects of genotype (*F*_(1,25)_ = 3.17; *p* > 0.05), sex (*F*_(1,25)_ = 1.42; *p* > 0.05) or genotype x sex interaction (*F*_(1,25)_ = 0.01; *p* > 0.05; Figure 2E), suggesting that mice in all groups showed comparable maze exploration.

### Object-based attention

We used the object-based attention test (OBA) to evaluate attention (McCarthy et al., 2018; McCarthy et al., 2020). There was no significant effect of genotype (*F*_(1,24)_ = 0.39; *p* > 0.05), sex (*F*_(1,24)_ = 0.02; *p* > 0.05), or genotype x sex interaction (*F*_(1,24)_ = 3.32; *p* > 0.05) on recognition index, a measure of attention, suggesting lack of differences in object-based attention among the different groups (Figure 2F).

### Tissue neurotransmitter concentration

Following the behavioral analyses, neurotransmitter content in the prefrontal cortex, medial prefrontal cortex, hippocampus, and dorsal striatum was analyzed. Striatal GABA tissue concentration showed a significant effect of sex (Table 2; male *<* female) and significant genotype x sex interaction (Table 2). Genotype did not produce significant effects (Table 2). *Post hoc* contrast analysis showed a significant decrease in striatal GABA tissue concentration in *Fmr1* KO males compared to WT males (*t*_(1,11)_ = −2.97, *p* < 0.05; Table 2). Tissue concentrations of GABA in the other brain regions did not show significant effects of genotype, sex, or genotype x sex interaction (Table 2).

**Table 2:** Brain tissue concentrations of neurotransmitters.

Neurotransmitter	Genotype	Sex	Genotype x sex
GABA			
Striatum	ns	*F*_(1,11)_ = 6.6^*^ (M < F)	*F*_(1,11)_ = 4.9^*^ (*Fmr1* KO M < WT M)
Prefrontal cortex	ns	ns	ns
Medial prefrontal cortex	ns	ns	ns
Hippocampus	ns	ns	ns
Glutamate			
Striatum	ns	*F*_(1,11)_ = 18.3^**^ (M < F)	*F*_(1,11)_ = 14.5^**^ (*Fmr1* KO M < WT M)
Prefrontal cortex	ns	ns	ns
Medial prefrontal cortex	ns	ns	ns
Hippocampus	ns	ns	ns
Serotonin			
Striatum	*F*_(1,11)_ = 5.4^*^ (*Fmr1* KO < WT)	ns	ns
Prefrontal cortex	ns	ns	ns
Medial prefrontal cortex	ns	ns	ns
Hippocampus	ns	ns	ns
Dopamine			
Striatum	ns	*F*_(1,11)_ = 10.8^**^ (M < F)	ns
Prefrontal cortex	ns	ns	ns
Medial prefrontal cortex	ns	ns	ns
Hippocampus	ns	*F*_(1,11)_ = 13.8^**^ (M < F)	ns
Noradrenaline			
Striatum	ns	ns	ns
Prefrontal cortex	ns	ns	ns
Medial prefrontal cortex	ns	*F*_(1,12)_ = 4.9^*^ (M < F)	ns
Hippocampus	ns	*F*_(1,11)_ = 9.5^*^ (M < F)	ns

^*^p < 0.05 and ^**^p < 0.01. ns, non-significant; M, male; F, female. Neurotransmitter concentrations in multiple brain regions were compared between male and female *Fmr1* KO and WT mice. A mixed model ANOVA was used to test the statistical significance of the effects of genotype, sex, and interactions between the two.

Changes in striatal glutamate tissue concentration showed the same pattern as that of GABA. There was a significant effect of sex (male < female) and a significant genotype x sex interaction (Table 2). Genotype did not produce significant effects. *Post hoc* contrast analysis showed a significant decrease in striatal glutamate tissue concentration in *Fmr1* KO males compared to WT males (*t*_(1,11)_ = −3.81, *p* < 0.01; Table 2). Tissue concentrations of glutamate in the other brain regions did not produce significant effects of genotype, sex, or genotype x sex interaction (Table 2).

Striatal serotonin tissue concentration showed significant effects of genotype (Table 2; *Fmr1* KO < WT), and no significant effect of sex or genotype x sex interaction (Table 2). Tissue concentrations of serotonin in the other brain regions did not show significant effects of genotype, sex, or genotype x sex interaction (Table 2).

Dopamine tissue concentration showed significant effects of sex in the striatum and the hippocampus (male < female; Table 2). The effects of genotype or genotype x sex interaction were not significant in the striatum or the hippocampus. The prefrontal cortex or the medial prefrontal cortex did not show significant effects of genotype, sex, or genotype x sex interaction (Table 2).

Noradrenaline tissue concentration showed a significant effect of sex in the medial prefrontal cortex and the hippocampus (male < female; Table 2). The effects of sex or genotype x sex interaction were not significant in the medial prefrontal cortex and the hippocampus. The striatum or the prefrontal cortex did not show significant effects of genotype, sex, or genotype x sex interaction (Table 2).

In summary, striatal GABA and glutamate content was reduced significantly in male *Fmr1* KO mice compare to wild type males. Striatal serotonin showed a significant effect of genotype, with lower concentrations in *Fmr1* KO mice. Dopamine tissue concentration showed significant effects of sex in the striatum and hippocampus, with lower concentrations in males compared to females regardless of genotype. Similarly, noradrenaline tissue concentration showed a significant effect of sex in the prefrontal cortex and the hippocampus, with lower concentrations in males compared to females.

### Testes and adrenal gland weights

Since males with fragile X show macroorchidism and increases in adrenal weights (Hagerman et al., 2017; Bhattacharya and Klann, 2012; Qin et al., 2011), we analyzed both these parameters in male and female mice. There was a significant increase in testes weight in *Fmr1* KO mice compared to WT counterparts (*t* = 3.245, df = 10; *p* < 0.01). Adrenal weights showed a significant effect of genotype (*Fmr1* KO > WT: *F*_(1,12)_ = 13.14; *p* < 0.001;) and sex (female > male: *F*_(1,12)_ = 1.42; *p* < 0.05;) but no significant genotype x sex interaction (*F*_(1,12)_ = 3.88, *p* > 0.05).

### RNA sequencing

Our behavioral and neurotransmitter analyses covered a broad range of functional (behavioral) and structural (brain regions for neurotransmitter tissue content analysis) domains. Since the nucleus accumbens of the ventral striatum is at the crossroads of multiple limbic circuits, we reasoned that gene expression in the ventral striatum could offer valuable insights into changes in a broad array of functional domains. Moreover, analysis of gene expression in the brain’s limbic centers such as the ventral striatum had not been undertaken in the *Fmr1* KO mouse model, despite reports of changes in motivation and reward mechanisms in FXS (Smith et al., 2014; Huebschman et al., 2021).

We identified 1,234 differentially expressed genes (DEGs) in the *Fmr1* KO mice relative to WT, with 576 genes upregulated and 658 genes downregulated. When we analyzed the data separately for male and female mice, 809 DEGs were found in males, with 375 genes upregulated and 434 genes downregulated. In female mice, we found 347 DEGs, with 119 genes upregulated and 228 genes downregulated (Table 3).

**Table 3:** Transcriptome analysis in the ventral striatum.

	DEGs	Up in *Fmr1* KO	Down in *Fmr1* KO
WT vs. *Fmr1* KO male + female	1,234	576	658
WT vs. *Fmr1* KO male only	809	375	434
WT vs. *Fmr1* KO female only	347	119	228

Number of differentially expressed genes (DEGs) as well as up-and down-regulated genes in the ventral striatum of *Fmr1* KO mice compared to WT mice.

As anticipated, *Fmr1* was the top downregulated DEG in both male and female mice. These findings were validated by qPCR analysis of *Fmr1* mRNA expression in the striatum, which showed very low but detectable levels in the *Fmr1* KO mice compared to WT mice (Supplementary Figure S2A). Western blot showed that FMRP was not detectable at all in either male or female *Fmr1* KO mouse striatum (Supplementary Figure S2B). WT male and female mice showed robust *Fmr1* mRNA and FMRP expression in the striatum.

### Gene set enrichment analyses

**Figure 4:**
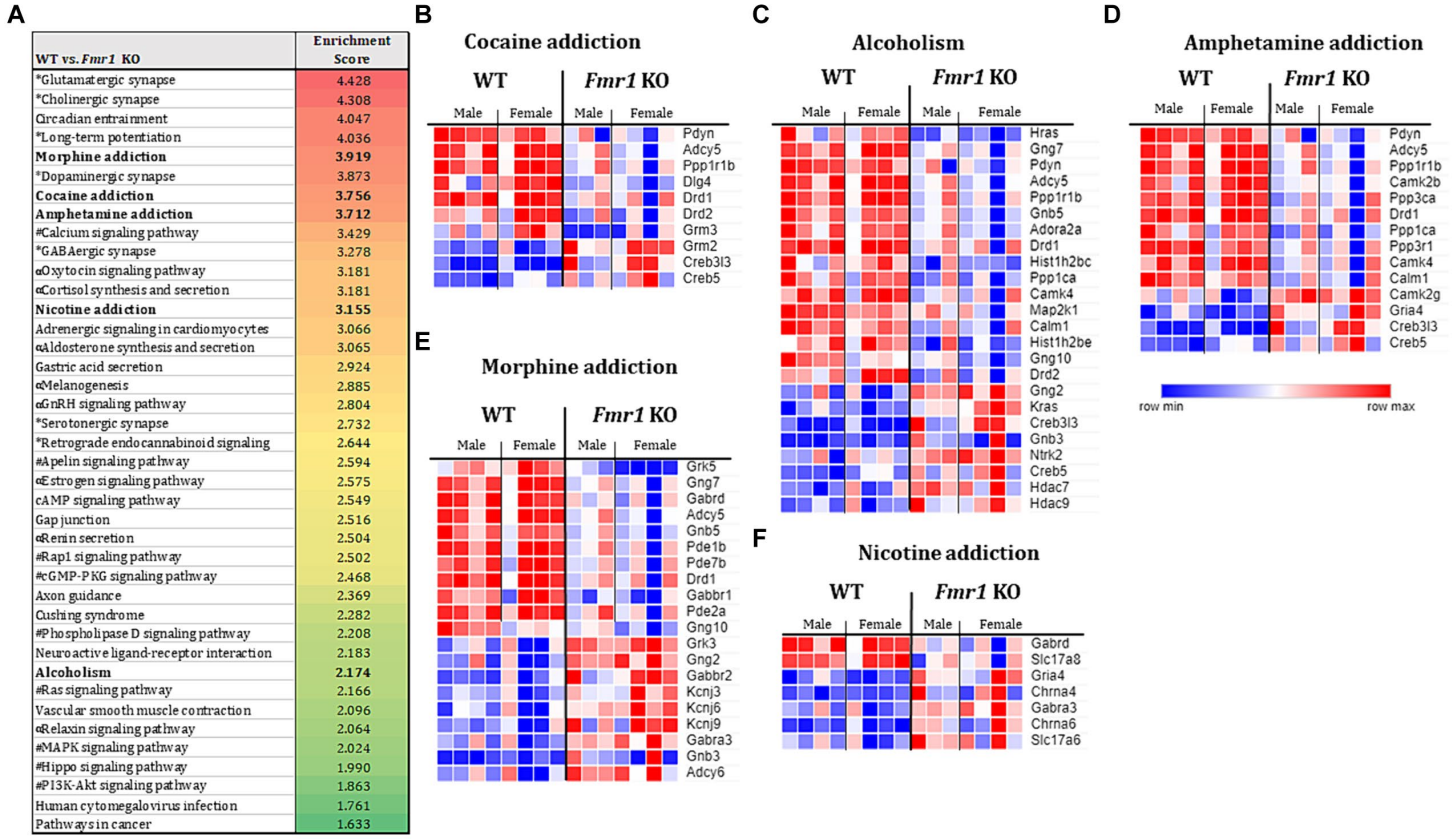
Transcriptome profiling of the ventral striatum by RNA sequencing in WT and *Fmr1* KO mice. KEGG gene set enrichment analysis of 1,243 differentially expressed genes (DEGs) in WT and *Fmr1* KO mice (data from males and females were combined) identified 40 significantly enriched pathways **(A)**. The pathways fall into 4 main categories (unique symbol for each pathway in **A**) namely, the nervous system (*); substance dependence (bold font) signal transduction (#), and the endocrine system (α). Heat map of normalized expression values of DEGs for male and female WT (male *n* = 4, female *n* = 4) and *Fmr1* KO (male *n* = 3, female *n* = 4) (columns) in each of the 5 substance dependence categories namely, cocaine addiction **(B)**, alcoholism **(C)**, amphetamine addiction **(D)**, morphine addiction **(E)**, and nicotine addiction **(F)**. The dark blue to dark red gradient represents gradient of minimum to maximum expression, respectively **(B–F)**.

The KEGG enrichment analysis of the 1,234 DEGs (Figure 4A) revealed 40 significantly enriched pathways. The vast majority (75%) of the total number of DEGs contributed to only 4 categories namely, nervous system, substance dependence, signal transduction, and endocrine system. Among the nervous system associated pathways, neurotransmission (e.g., glutamatergic synapse, GABAergic synapse, cholinergic synapse, dopaminergic synapse, serotonergic synapse, norepinephrine signaling) was prominently represented.

Consistent with the KEGG pathway analysis, phenotype set enrichment analysis of the 1,234 DEGs identified drug reward pathways as being significantly enriched. Among the enriched reward pathways, conditioned place preference and behavioral response to morphine were at the very top of the list. In addition, multiple pathways associated with neurotransmission (e.g., synaptic transmission, and increased susceptibility to neuronal excitotoxicity, abnormal glutamate-mediated receptor currents, and abnormal excitatory postsynaptic currents pathways) were identified as well (Supplementary Figure S3).

Evaluation of gene expression in the cocaine addiction, alcoholism, and amphetamine addiction pathways for male and female WT and *Fmr1* KO mice revealed that approximately 30% of the DEGs were upregulated and 70% were downregulated in the *Fmr1* KO mice (Figures 4B–D). Among the downregulated DEGs *Pdyn* (prodynorphin), *Adyc5* (adenylate cyclase 5), *Ppp1r1b* (protein phosphatase 1 regulatory inhibitor subunit 1B) and *Drd1* (dopamine receptor D1) were common to all 3 pathways. Among the upregulated DEGs, *Creb5* (cAMP responsive element binding protein 5) and *Creb3l3* (cAMP responsive element binding protein 3 like 3) were common to all 3 pathways (Figures 4B–D). Evaluation of gene expression in the nicotine addiction pathway revealed gene expression changes that were opposite to those in the other 3 pathways (Figure 4F). Specifically, approximately 30% of the DEGS were downregulated and 70% were upregulated in the nicotine addiction pathway. In the morphine addiction pathway, approximately equal proportion (50%) of the DEGs were upregulated and downregulated (Figure 4E).

## Discussion

Our data show that *Fmr1* KO mice show significant changes in locomotor activity, approach-avoidance behavior, motor impulsivity, and nest building behavior. Striatal GABA, glutamate and serotonin tissue concentrations are significantly decreased in the *Fmr1* KO mice. RNA sequencing of the ventral striatum identified 1,234 DEGs in the *Fmr1* KO mice relative to WT mice. Sex differences were evident in nest building behavior, striatal neurotransmitter content, and the number of differentially expressed genes in the ventral striatum.

There are significant genotypic differences between humans with FXS and the *Fmr1* KO mouse model used here, especially between males and females. In FXS, the affected males carry the CGG expansion in the mutant *FMR1* gene on the X chromosome and lack a “normal” copy of *FMR1*. FXS carrier females have a mutant and a normal *FMR1* allele each on their X chromosomes. Although rare, women may carry two copies of the mutant *FMR1* gene (one on each X chromosome) (Vafaeie et al., 2021). In the *Fmr1* KO mouse model used here, the *Fmr1* gene is deleted in both males and females. We show that *Fmr1* mRNA expression (Supplementary Figure S2A) was virtually absent (only low levels were expressed) (Yan et al., 2004) and FMRP expression was undetectable in the striatum (Supplementary Figure S2B) in both sexes of the *Fmr1* KO mouse. Thus, genotype differences between humans with FXS and the *Fmr1* KO mouse should be considered while extrapolating data from the *Fmr1* KO mouse to humans with FXS.

Our findings from the behavioral assays are largely consistent with previous reports that used the *Fmr1* KO mouse model used here. For example, our findings of increased spontaneous exploratory activity and locomotor activity in the *Fmr1* KO mice are consistent with findings from multiple previous studies, although the methods used for the analysis differed among the studies (Bakker et al., 1994; Dahlhaus and El-Husseini, 2010; Spencer et al., 2005; Peier et al., 2000; Pietropaolo et al., 2011; Ding et al., 2014; Gholizadeh et al., 2014; Uutela et al., 2014; Mineur et al., 2002; Liu et al., 2011; Longo et al., 2023).

The elevated plus maze assay revealed significant increases in time spent in the open arms and the number of entries into the open arms in the *Fmr1* KO mice, consistent with findings from previous reports (Arsenault et al., 2016; Dansie et al., 2013; Sare et al., 2016). We interpret this outcome as evidence of increased approach behavior (Buck et al., 2019), suggestive of novelty-seeking and risk-taking (Cloninger, 1986; Hansson et al., 2012; Kelley et al., 2004; Wang et al., 2015). Others interpreted these outcomes as evidence of reduced anxiety (Hagerman et al., 2017; Spencer et al., 2005; Schaefer et al., 2017). Further studies would be necessary to discriminate between approach-avoidance and anxiety-like behaviors in the *Fmr1* KO mice.

The cliff avoidance reflex assay revealed motor impulsivity in the *Fmr1* KO mice consistent with previous reports in the literature (Moon et al., 2006). The *Fmr1* KO mice were 10 times more likely to fall from the platform compared to the WT mice, as indicated by their poor cliff avoidance reflex score. It is possible that the increased number of falls are the result of the increased spontaneous locomotor activity, exploratory activity or approach behavior in the *Fmr1* KO mice rather than the result of motor impulsivity *per se*. Our data do not discriminate between these possibilities fully. However, the *Fmr1* KO mice did not show increased arm entries in the Y-maze (Figure 2E) nor did they show increased locomotor activity during nest building in the novel environment (Supplementary Figure S4). In other words, the increased locomotor activity, exploratory activity, or approach behavior did not transfer to these behavioral assays. Therefore, we suggest that the outcome from the cliff avoidance reflex assay reflects motor impulsivity in the *Fmr1* KO mice.

Individuals with FXS are often diagnosed with social phobia and social interaction deficits and some of these behaviors have been recapitulated in the *Fmr1* KO mouse model, although findings from the different studies are variable (Dahlhaus and El-Husseini, 2010; Spencer et al., 2005; Pietropaolo et al., 2011; Mineur et al., 2002; Liu et al., 2011; McNaughton et al., 2008). We used the tube test of social dominance, which has been used to study social hierarchy in mice (Fan et al., 2019). We found that *Fmr1* KO mice lost significantly more matches when paired against age- and sex-matched WT partners in the tube test and were 14 times more likely to lose a match regardless of sex, suggesting a significant impairment of social dominance. These findings are consistent with previous reports of impaired social dominance in the *Fmr1 KO* mice (Spencer et al., 2005; Pacey et al., 2011).

We found significant deficits in nest building behavior in the *Fmr1* KO mice in the home as well as novel environments, consistent with findings from previous studies (Gurney et al., 2017; Carreno-Munoz et al., 2018; Gross et al., 2019; Gross et al., 2015). Nest building is a motivated behavior displayed by both sexes of multiple species. Poor quality of the nest or delay in building a fully formed nest reflect deficits in motivated behaviors and arousal-anxiety disequilibrium (Jacobson et al., 2020). The environment of the novel cage introduces an element of anxiety, which the mice must overcome to build the nest. Limbic circuits involving the hippocampus and ventral striatum, which influence motivated behaviors as well as emotional behaviors including anxiety, play a role in the regulation of nest building behavior (Deacon et al., 2002; Luo et al., 2018). The specific contributions of motivation, reward, and anxiety-arousal to nest building are difficult to distinguish and the contribution of each to the overall deficit in nest building in the *Fmr1* KO mouse is difficult to establish using the nest building assay alone. Additional studies will be necessary to address these issues.

The poor nest building and the increased “approach” behavior (or reduced anxiety-like behavior) exhibited by the *Fmr1* KO mice in the elevated plus maze assay could have common origins. The nest building behavior likely reflects intrinsic motivation to create a place of safety, warmth and protection. If one conceded the speculative interpretation that the poor nest building by the *Fmr1* KO mice reflects “disregard” for safety, then such behavior would be consistent with the increased “approach” behavior in the elevated plus maze. We emphasize that additional research would be needed to test the validity of this suggestion.

We did not find significant differences between *Fmr1* KO and WT mice in spatial working memory or object-based attention. There are reports of significant deficits in working memory and attention in the *Fmr1* KO mice, although a consensus is yet to emerge (review in Kazdoba et al., 2014). Methodological differences may contribute to the variability in outcomes.

A principal goal of the present study was an investigation of sex differences in the behavioral traits in the *Fmr1* KO mouse model. Selective analysis of male mice is frequent in FXS research (Spencer et al., 2005; Arsenault et al., 2016; Dansie et al., 2013; Sare et al., 2016; Pacey et al., 2011). However, when both sexes of mice are examined, as in the present study, lack of sex differences in behavioral parameters is a frequent finding (Bakker et al., 1994; Ding et al., 2014; Gholizadeh et al., 2014; Gauducheau et al., 2017; Schmitt et al., 2022). Consistent with this observation, a comparison between male and female mice showed that most of the behavioral traits analyzed here did not show a sex difference. Nest building in the novel environment was the only behavior that showed significant effects of sex x genotype. The nest building deficits in the novel environment were more pronounced and emerged earlier in the task in male *Fmr1* KO mice compared to females. Nest building deficits in male *Fmr1* KO mice were reported by others, although these reports did not study female mice (Gurney et al., 2017; Carreno-Munoz et al., 2018; Gross et al., 2019; Gross et al., 2015).

Analysis of neurotransmitter content showed significant deficits in striatal GABA and glutamate content in male *Fmr1* KO mice, and deficits in serotonin content in male and female *Fmr1* KO mice demonstrating significant sex differences. Previous studies reported an overall reduction in GABA tone in FXS patients as well as *Fmr1* KO mice based on analysis of GABA synthesis, release, and GABA receptor signaling mechanism (for review see Paluszkiewicz et al., 2011; Dionne and Corbin, 2021). Reduced glutamatergic neurotransmission has not been reported in FXS patients. However, metabotropic glutamate receptor antagonists produce behavioral improvements in *Fmr1* KO mouse models (Yan et al., 2005; Xu et al., 2012; Osterweil et al., 2010), which implies increased rather than decreased glutamatergic tone in the *Fmr1* KO mouse brain. In some patients with FXS, lack of FMRP may affect serotonin-mediated pathways (Hanson and Hagerman, 2014; Protic et al., 2019) and serotonin receptor agonists and selective serotonin reuptake inhibitors have been shown to ameliorate behavioral deficits in the *Fmr1* KO mouse (Uutela et al., 2014; Costa et al., 2018; Tao et al., 2023; Kim et al., 2022). The deficits in striatal glutamate, GABA and serotonin likely contribute to the behavioral changes observed in the *Fmr1* KO mice, although a correlation with each behavioral trait is difficult to establish. Further study to characterize potential changes in pharmacological targets of neurotransmission such as receptor signaling, neurotransmitter synthesis and re-uptake could offer direct translational implications for FXS.

KEGG and phenotype gene set enrichment analyses identified signal transduction, nervous system, and endocrine system categories were significantly enriched in the *Fmr1* KO mouse ventral striatum (Figure 4). Consistent with data from the tissue neurotransmitter content assays, neurotransmission (e.g., glutamatergic synapse, GABAergic synapse, cholinergic synapse, dopaminergic synapse, serotonergic synapse, norepinephrine signaling) category was prominently represented among the enriched pathways.

Gene set enrichment analysis further highlighted substance dependence and drug reinforcement as significantly enriched pathways in the *Fmr1* KO ventral striatum. Cocaine, amphetamine, nicotine, morphine, and alcohol addiction pathways were among the significantly enriched. Downregulated DEGs such as *Pdyn*, *Ppp1r1b*, *Drd1*, and *Adcy5*, which are known to be associated with reward and addiction (Berman et al., 2014; Kim et al., 2006; Dichter et al., 2012; Baik, 2013; McLaughlin et al., 2003; Galeote et al., 2009) were found in the cocaine, alcohol, amphetamine, and morphine addiction pathways in the *Fmr1* KO ventral striatum. Previous reports showed that *Adcy5* KO mice have reduced reward response to morphine, whereas *Ppp1r1b* (commonly referred to as Darpp32) KO mice have reduced responses to cocaine in conditioned place preference test (Kim et al., 2006; Zachariou et al., 2002).

Other reports in the literature demonstrate reduced cocaine reward, behavioral sensitization, and cocaine-seeking behavior in *Fmr1* KO mice (Smith et al., 2014; Huebschman et al., 2021). These behavioral changes are associated with increased dendritic spine density and synaptic strength in the nucleus accumbens (Smith et al., 2014) a major constituent of the ventral striatum. A recent study showed that *Fmr1* mRNA is reduced in leukocytes of patients diagnosed with alcohol or drug dependence in comparison to healthy controls (Krasteva et al., 2020), further demonstrating a potential link between *Fmr1* and drug dependence. The present study offers evidence from gene set enrichment analysis that compromised motivation and drug reward mechanisms are likely an intrinsic property in the *Fmr1* KO mouse. We did not seek direct evidence of compromised motivation or drug reinforcement in the *Fmr1* KO mice.

Finally, we did not observe significant differences in litter size or the age of acquisition of developmental milestones in the *Fmr1* KO offspring (Table 1). There were no significant differences in body weight at any time during development (Table 1) or in adulthood between the *Fmr1* KO and WT mice. Thus, in the present study, the mouse model did not recapitulate findings of developmental delays in FXS nor did they support the observation that children and adolescents with FXS are heavier than age-matched controls (McLennan et al., 2011). We found that the testes and adrenal gland weights (as a percentage of total body weight) were increased in the *Fmr1* KO mice, consistent with previous reports (Hagerman et al., 2017; Bhattacharya and Klann, 2012; Qin et al., 2011). Both male and female *Fmr1* KO mice had increased adrenal gland weights compared to WT mice. Incidentally, females had greater adrenal gland weights than males regardless of genotype (Bielohuby et al., 2007).

In summary, our findings on behavioral traits in the *Fmr1* KO mouse agree with those from earlier studies. We identified significant deficits in striatal GABA, glutamate and serotonin content in the *Fmr1* KO mouse and significant changes in gene expression in the *Fmr1* KO mouse ventral striatum. Our methodologies do not permit us to draw direct relationships among the changes observed in the different parameters, although a general relationship between deficits in striatal neurotransmitter content, enrichment of the neurotransmission category in gene set enrichment pathways and behaviors such as locomotor hyperactivity, approach behavior, motor impulsivity, social dominance and nest building appears plausible. The enrichment of pathways of motivation and drug reward in the ventral striatal gene set enrichment analysis offers novel perspectives on compromised motivation and reward mechanisms in the *Fmr1* KO mouse. Although these findings do not offer insights into the direction of the change (up- or down-regulation), previous reports (Smith et al., 2014; Huebschman et al., 2021) suggest downregulation of motivation and reward processing in FXS, consistent with current ideas of dysfunction in these mechanisms in autism spectrum disorder (Keifer et al., 2021). Finally, sex differences in nest building behavior in novel environments, striatal neurotransmitter content, and gene expression in the ventral striatum highlight the significance of including male and female *Fmr1* KO mice in FXS research.

## Data Availability

The data presented in the study are deposited in the NCBI Gene Expression Omnibus repository, https://www.ncbi.nlm.nih.gov/geo/query/acc.cgi?acc=GSE275170; accession number GSE275170.

## Footnotes

^1^ https://www.jax.org/strain/000664

^2^ https://www.jax.org/strain/003025

^3^ http://www.bioinformatics.babraham.ac.uk/projects/fastqc/

^4^ http://www.ncbi.nlm.nih.gov/geo/
