# Supplementary material for: Behavioral, neurotransmitter and transcriptomic analyses in male and female Fmr1 KO mice
Source: Front Behav Neurosci. 2024 Sep 6;18:1458502. doi: 10.3389/fnbeh.2024.1458502 (PMC11412825; doi:10.3389/fnbeh.2024.1458502)
Supplement: Supplementary file 1 [file Data_Sheet_1.PDF]

A. Righting Reflex

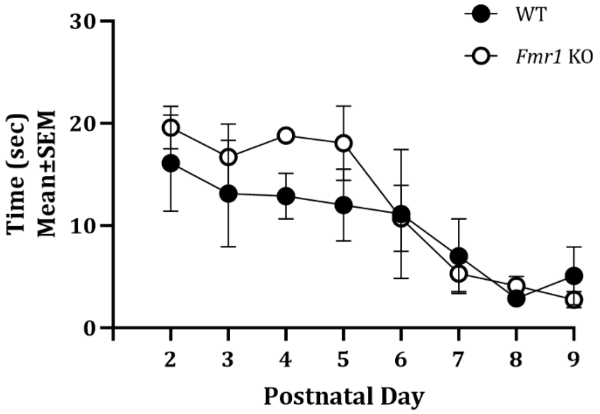

B. Negative Geotaxis

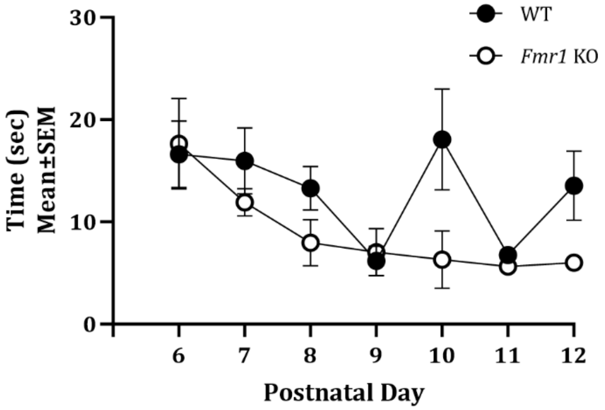

C. Cliff Avoidance

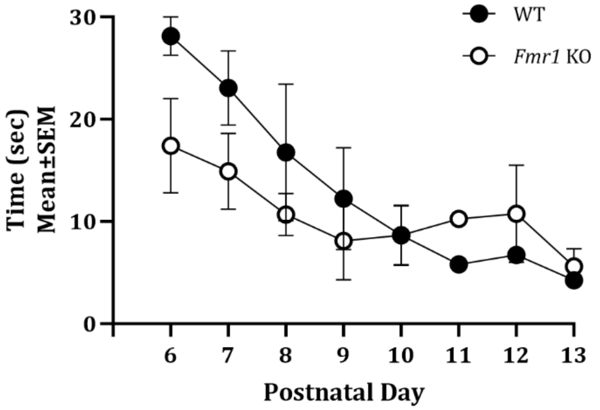

Supplemental Figure 1: Neuromotor skills in preweaning mice. Righting reflex **(A)** negative geotaxis **(B)** and cliff avoidance in WT and *Fmr1* KO mice. Time taken (seconds) to complete each task daily. A mixed model ANOVA showed no significant effects of genotype. Mean±SEM, n=3-4 litters per genotype.

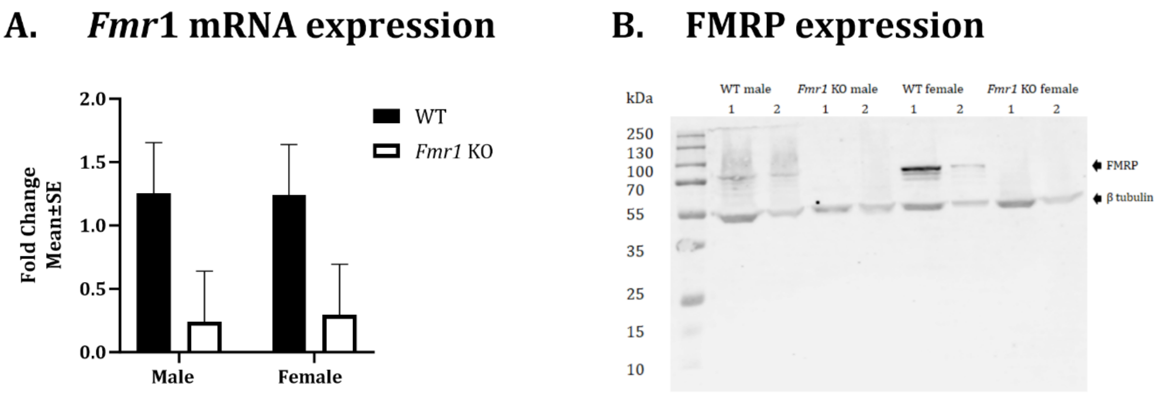

Supplemental Figure 2: Striatal *Fmr1* mRNA and FMRP expression in male and female *Fmr1* KO mice. mRNA fold change measured by qPCR for *Fmr1* was lower in the *Fmr1* KO compared to the WT in male and female striatum (Mixed model ANOVA; Genoytype: $F_{(1,8)}$=5.99, p<0.05;*Fmr1* KO < WT; **A).** FMRP protein expression was undetectable in the striatum of *Fmr1* KO male and female mice **(B)**. n=2-3 per genotype per sex.

WT vs. *Fmr1* KO	Enrichment Score
impaired conditioned place preference behavior	7.2
impaired behavioral response to morphine	6.9
abnormal conditioned place preference behavior	6.3
impaired behavioral response to xenobiotic	5.3
decreased urine creatinine level	5.3
impaired behavioral response to addictive substance	5.1
decreased chemically-elicited antinociception	4.8
abnormal chemically-elicited antinociception	4.4
abnormal mammary gland lobule morphology	4.4
abnormal behavioral response to addictive substance	4.2
reduced long term depression	4.1
increased susceptibility to neuronal excitotoxicity	3.9
abnormal long term depression	3.5
abnormal glutamate-mediated receptor currents	3.2
impaired conditioning behavior	3.2
abnormal spatial reference memory	3.0
abnormal behavioral response to xenobiotic	2.9
abnormal excitatory postsynaptic currents	2.8
abnormal synaptic depression	2.8
abnormal depression-related behavior	2.6
abnormal aggression-related behavior	2.5
decreased anxiety-related response	2.4
abnormal action potential	2.4
abnormal associative learning	2.4
abnormal anxiety-related response	2.4
increased anxiety-related response	2.3
abnormal parental behavior	2.2
abnormal fear/anxiety-related behavior	2.2
abnormal seizure response to inducing agent	2.2
abnormal pain threshold	2.1
abnormal spatial learning	2.1
abnormal CNS synaptic transmission	2.1
abnormal touch/ nociception	2.1
abnormal long term potentiation	2.1
abnormal startle reflex	2.1
abnormal synaptic transmission	2.0
abnormal nervous system electrophysiology	1.9
abnormal learning/memory/conditioning	1.9
abnormal cognition	1.9
hyperactivity	1.8
abnormal social/conspecific interaction	1.8
abnormal neuron physiology	1.8
abnormal emotion/affect behavior	1.8
abnormal sensory capabilities/reflexes/nociception	1.7
abnormal reflex	1.7
abnormal nervous system physiology	1.5
abnormal involuntary movement	1.5
abnormal locomotor activation	1.5
abnormal locomotor behavior	1.4
abnormal voluntary movement	1.4

Supplemental Figure 3: Phenotype set enrichment analysis of the ventral striatum in WT and *Fmr1* KO mice. Phenotype set enrichment analysis of differentially enriched genes (DEGs) in the ventral striatum of WT and *Fmr1* KO mice (1,234 DEGs, male and female combined) identified 50 significantly enriched pathways. Impaired conditioned place preference behavior and impaired behavioral response to morphine were at the top of the list, which is ordered by high to low enrichment score. Overall, the findings were in agreement with the findings from the KEGG enrichment analysis of the DEGs (Figure 4).

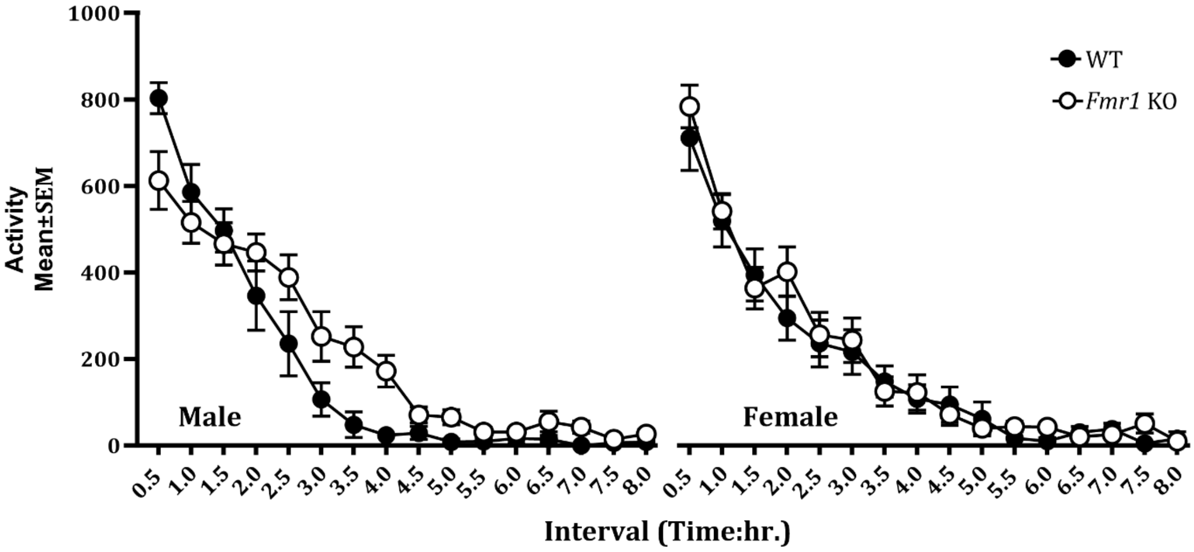

Supplemental Figure 4: Locomotor activity in WT and *Fmr1* KO mice in the novel environment. Locomotor activity was recorded at 30- minute intervals during the nest building assay in the novel environment over a period of 8 h in the Photobeam activity system (San Diego Instruments). A mixed model ANOVA showed no significant effects of genotype or genotype x sex interaction. Data from male and female mice are shown separately for each genotype. n=7-15 per genotype per sex.
